# Rationale and design of the PLACID study: a randomised trial comparing the efficacy and safety of inhaled loxapine versus IM aripiprazole in acutely agitated patients with schizophrenia or bipolar disorder

**DOI:** 10.1186/s12888-017-1291-5

**Published:** 2017-04-04

**Authors:** L. San, G. Estrada, N. Oudovenko, E. Vieta

**Affiliations:** 1 Mental Health Department, Parc Sanitari Sant Joan de Deu, CIBERSAM, Carrer Camí Vell de la Colonia, 25 Sant Boi de Llobregat, Barcelona, Spain; 2 Ferrer, Barcelona, Spain; 3 Hospital Clínic, Institute of Neuroscience, University of Barcelona, IDIBAPS, CIBERSAM, Barcelona, Spain

**Keywords:** Acute agitation, Schizophrenia, Bipolar disorder, Antipsychotic, Inhaled-loxapine, IM-aripiprazole, Rapid onset

## Abstract

**Background:**

The management of acute agitation manifesting in patients with schizophrenia or bipolar disorder requires swift pharmacological intervention to provide rapid symptomatic relief and prevent escalation to aggression and violence. Antipsychotic medications are widely used in this setting and the availability of an inhaled formulation with deep lung absorption of the antipsychotic loxapine has the potential to deliver a faster onset of therapeutic effect than the available intramuscular formulations of antipsychotics.

**Methods:**

The efficacy of inhaled loxapine and the alternative antipsychotic aripiprazole delivered via intramuscular (IM) injection will be compared in the Phase IIIb PLACID study. Adults (18–65 years) with a confirmed diagnosis of schizophrenia or bipolar I disorder presenting with acute agitation will be randomly assigned to open-label treatment in a 1:1 ratio. Clinical evaluation will be conducted by raters blinded to treatment assignment. The primary efficacy endpoint is time to response (defined as a Clinical Global Impression of Improvement [CGI-I] score of 1 [very much improved] or 2 [much improved]).

Secondary endpoints will include the percentage of responders at different time points after dosing; the proportion of patients who receive 1 or 2 doses of study drug; time to second dose; time to rescue medication; satisfaction with study drug (evaluated using Item 14 of the Treatment Satisfaction Questionnaire for Medication); and safety and tolerability. Approximately 360 patients will be recruited with an interim analysis conducted once 180 patients have completed the study to decide whether to stop for futility or continue with or without an increase in the sample size up to additional 288 patients.

**Discussion:**

The PLACID trial will assess the efficacy and safety of inhaled loxapine with deep lung absorption compared with the IM antipsychotic, aripiprazole, in acutely agitated patients with schizophrenia or bipolar disorder. In the event that the median time to response of inhaled loxapine is significantly shorter than that of the intramuscular aripiprazole, the PLACID study has the potential to support the inhaled antipsychotic therapy as the standard of care in this setting.

**Trial registration:**

The study protocol was registered with the European Clinical Trials Database on the 31 October 2014 (EudraCT number 2014–000456-29).

**Electronic supplementary material:**

The online version of this article (doi:10.1186/s12888-017-1291-5) contains supplementary material, which is available to authorized users.

## Background

Schizophrenia and bipolar disorder are estimated to affect 21 million and 60 million individuals worldwide, respectively [1]. Agitation, defined as a state of cognitive and motor hyperactivity characterized by excessive or inappropriate motor or verbal activity with an emotional arousal element, is common among patients with schizophrenia or bipolar disorder [2–4]. For these patients, the manifestation of acute agitation represents a serious, disruptive and morbid complication that can escalate rapidly and unpredictably from distress to loss of control and physical aggression and violence towards themselves, other patients and their carers [4–6].

Acute agitation is frequently encountered in both medical and psychiatric emergency settings and should be managed initially with verbal de-escalation and environmental management [7, 8]. Continued escalation of agitation may ultimately require physical restraint and pharmacological treatment [7, 8]. However, such coercive management measures may damage the therapeutic relationship between patients and their carer’s and evoke feelings of panic, fear, powerlessness, anger, frustration, and injustice [2]. The consequence of this negative association of feelings may destroy trust and lead to patient reluctance to seek psychiatric care [3]. Early intervention for acute agitation with a medication delivered via a non-traumatic route has the potential to avoid the negative feelings evoked by a traumatic therapeutic encounter and reinforce the partnership between patient and their carer [2, 3].

The costs associated with agitation, especially agitation that escalates to aggression and violence, among patients with psychiatric disorders may also be considerable although precise estimates of the overall burden are lacking. When inadequately managed, agitation among psychiatric patients can result in an increase in the number and duration of inpatient hospital stays, a reduced likelihood of discharge to community and use of coercive measures and/or escalation to patient violence, presenting a substantial economic burden to the healthcare system [8, 9]. In Spain, the annual cost of psychiatric mechanical restraint has been estimated at €27 million [9]. A separate study conducted in the UK, estimated that the annual cost per psychiatric ward for containment was €267,069 in 2014 [10].

Agitation among patients with psychiatric disorders requires early recognition and swift intervention with treatments that can achieve rapid symptom relief and avoid the progression of agitation to aggression and violence [7]. The acute treatment of agitation associated with schizophrenia or bipolar disorder includes pharmacological tranquilization with oral antipsychotic agents given alone or in combination with benzodiazepines [11–13]. However, while a preferred route of delivery in terms of maintaining a non-traumatic experience for the patient, orally dosed agents may not be optimal in terms of delivering a sufficiently rapid onset of therapeutic effect. Intramuscular (IM) formulations have been shown to provide relatively faster symptomatic relief compared with oral formulation of antipsychotics but have the disadvantage of a potentially traumatic experience for the patient [2, 14].

Inhaled loxapine with deep lung absorption is the first anti-agitation medication available to provide rapid control of agitation combined with a noncoercive non-invasive route of administration. It is administered by inhalation using the Staccato delivery system. The efficacy of inhaled loxapine for the management of agitation among patients with schizophrenia or bipolar I disorder has been confirmed in placebo-controlled, Phase III trials [15–17] and is supported by indirect comparisons with other antipsychotics [18]. However, head-to-head comparisons with other antipsychotics, including those delivered via IM injection, are lacking. Head-to-head trials, often neglected by regulatory authorities and industry sponsors, are important for clinical practice [19]. The PLACID study will compare inhaled loxapine and IM aripiprazole (considered a standard of care in this setting; [20]) for the prompt stabilization of acutely agitated patients with schizophrenia or bipolar disorder.

## Methods

The PLACID study will evaluate the efficacy, defined as time to response (Clinical Global Impression of Improvement [CGI-I] or 1 or 2) of inhaled loxapine as compared with aripiprazole administered via IM injection in acutely agitated patients with schizophrenia or bipolar disorder.

Inhaled loxapine is available in 10 mg dose, being the delivered dose 9,1 mg, a maximum of 2 doses can be administered during 24 h (24). For IM aripiprazole the standard dose is 9,75 mg with a possibility of two additional doses during 24 h (14). In this study a maximum of 2 doses of both study drugs are allowed [14, 21].

The study will be conducted at up to 30 centres across Europe including centres in the Czech Republic, Germany, Russia and Spain and will include male and female patients aged between 18 and 65 years with a confirmed diagnosis of schizophrenia or bipolar I disorder according to Diagnostic and Statistical Manual of Mental Disorders (DSM-5) criteria.

### Design

**Fig. 1:**
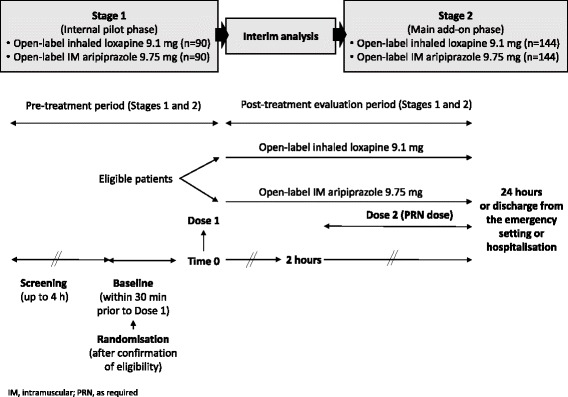
Study design

The PLACID study is a Phase IIIb, open-label, rater blind, randomised, active control, prospective, parallel group, multicentre clinical trial (Fig. 1, Table 1). The study will be conducted in two phases. The first phase will enrol approximately 180 patients who will be randomly assigned (in a 1:1 ratio) to open-label treatment. Clinical evaluation will be carried out by personnel blinded to treatment assignment. Patients will be instructed not to tell the blinded assessor which treatment they received.

**Table 1:** PLACID study flow

Procedure	Pre-treatment period		Post-treatment evaluation period									
	Screening^a^ *(up to 4 h)*	Baseline *(within 30 min prior to dose 1)*	Time 0					1 h		2 h	4 h	24 h or end of the agitation episode, whichever occurs first
			0 min	10 min	20 min	30 min	50 min	60 min	90 min	120 min	240 min	
Informed consent^b^	X											
Inclusion/exclusion criteria	X	X^c^										
Demographics	X											
Medical history	X											
Pre-dose medications	X^d^	X^d^										
Physical examination^e^	X											X
Urine pregnancy test (females of child-bearing potential)^f^	X											
Randomisation		X										
Study drug administration			X	← NOT ALLOWED →							Up to 1 dose PRN^g^	
CGI-S Scale^h^		X										
CGI-I Scale^h^				X ± 2 min	X ± 2 min	X ± 2 min	X ± 2 min	X ± 5 min	X ± 5 min	X ± 5 min		X ± 1 h
TSQM (Item 14)^h^										X		X
Vital signs^h,i^		X						X		X	X	X
AEs^j^			X→	→	→	→	→	→	→	→	→	X
Concomitant medications^k^	X→	→	→	→	→	→	→	→	→	→	→^k^	X^k^
Discharge from the study												X

*AE* adverse event, *CGI-I* Clinical Global Impression of Improvement, *CGI-S* Clinical Global Impression of Severity, *eCRF* electronic case report form, *HCG* human chorionic gonadotropin, *ICF* informed consent form, *PRN* pro re nata (as needed), *TSQM* Treatment Satisfaction Questionnaire for Medication. ^a^Patients must remain in the study centre during screening. ^b^The approved ICF must be signed by patients with consent capacity before completing any protocol-specific procedures. ^c^Inclusion criteria #3 and 4 and Exclusion criteria 4 and 12 will be assessed at baseline. ^d^All medications taken within 30 days of the study start, includind medications taken in the 24 hours prior to the first dose of study medication, should be recorded on the eCRF. ^e^Baseline and end-of-study examinations will be completed; other examination should be a focused physical examination at the discretion of the Investigator based on the patient's circumstances. ^f^Females of documented postmenopausal status and inpatients who previously tested negative for pregnancy (serum β-HCG) upon admission are not required to undergo the urine test at screening. ^g^Note that patients who only receive one dose of study drug (Dose 1) may not be given rescue medication (unless medically required). Rescue medication may be administered per the Investigator's judgement from 20 minutes after Dose 2 of the study medication has been given (and after the 2-hour efficacy assessments have been completed). ^h^At all time points, perform rating scales/ assessments in the following order, as applicable: CGI, TSQM (Item 14), vital signs. ^i^Pulse, systolic and diastolic blood pressure and respiraion rate will be recorded. ^j^AEs will be recorded for at least 24 hours after Dose 1 or the study medication or the end of the agitation episode as per the Investigator's judgement, whichever occurs first. Patients should be monitored for bronchospasm for => 1 hour after Dose 1 and Dose 2 (if Dose 2 is administered). ^k^Concomintant medications, including those to treat AEs, will include all medications that patients were already taking at the time of screening or started during the course of the study. If rescue medication is taken, the time that it is taken will be recorded. Whether rescue medication has been taken by 4 hours and by 24 hours after Dose 1 or the end of the agitation episode as per the Investigator's judgement, whichever occurs first, will be recorded

An interim analysis will be performed on the phase 1 data to determine whether to continue into phase 2 or to stop the trial due to futility or unfeasibility. The final sample size of the PLACID study will be re-calculated based on the interim analysis results. The two-phase design with interim analysis is justified due to the limited availability of CGI-I data during the first 2 h’ post-administration both for inhaled loxapine and IM aripiprazole, and therefore making difficult the sample size estimation. Data demonstrating meaningful improvements from baseline on the CGI-I scale at 120 mins post dose are available [15, 16, 20, 22] for both treatments, but data demonstrating the time course of these improvements during the first two hours of treatment, when rapid and effective action is required to minimise the risks associated with acute agitation, are lacking.

### Patients

The study population will consist of patients meeting DSM-5 criteria for schizophrenia or bipolar I disorder experiencing an acute episode of agitation, both hospitalized and attending to a psychiatric or general emergency room.

To be eligible to participate patients must be clinically agitated at baseline with a value ≥4 for the Clinical Global Impression of Severity (CGI-S; [23]) scale and otherwise in good health. Patients will be specifically excluded from participation if their agitation is judged to be caused primarily by acute alcohol or drug intoxication/withdrawal. Those patients judged to be at serious risk for suicide will also be excluded. Patients treated with benzodiazepines or other hypnotics or oral or short-acting IM antipsychotics within 1 h prior to study drug administration will be excluded although those patients may be subsequently reassessed for inclusion. Other exclusion criteria will include pregnancy or breastfeeding, a history of or current significant hepatic, renal, gastroenterologic, respiratory, cardiovascular, endocrinologic, neurologic or haematologic disease as well as acute or active airways disease.

All patients will be required to provide written informed consent prior to undergoing any study procedure. Patients considered by appropriately qualified staff to be of impaired capacity to provide informed consent will not be eligible for participation. In Spain a proxy or deferred informed consent is possible. If patients have recovered the capacity, they will be asked to provide the informed consent; otherwise the data will not be used in the analysis.

### Study treatments

Baseline evaluations will be performed as close as possible to, and within 30 min before, administration of initial study medication. Patients will be randomly assigned to receive either inhaled loxapine 10 mg (delivered dose 9.1 mg) using the hand-held, portable, *Staccato* delivery system or IM aripiprazole 9.75 mg per 1.3 mL dose. Patients will receive a maximum of 2 doses of either study drug with the second dose given if required at least 2 h after the first dose.

Randomisation will be achieved using an interactive voice response system and will be stratified according to the patient’s disease type (schizophrenia or bipolar I disorder).

The planned duration of participation in the study will be up to approximately 30 h consisting of a screening and baseline phase of up to 4.5 h and a post-treatment period which will commence with the administration of the first dose of study drug and will continue for at least 4 h and up to a maximum of 24 h.

### Outcomes and endpoints

The primary efficacy endpoint is time to response measured during the first 2 h after the first dose. Response is defined as a CGI-I score of 1 (very much improved) or 2 (much improved). Secondary endpoints will include: the proportion of patients achieving response at 10, 20, 30, 50, 60, 90 and 120 min after the first dose of study drug; the total number of patients who receive 1 or 2 doses of study drug without rescue medication by 4 h and 24 h after the first dose of study drug; time to rescue medication; time to the second dose of study drug; and satisfaction with study drug (evaluated using Item 14 of the Treatment Satisfaction Questionnaire for Medication [TSQM]; [24]). Safety and tolerability will also be assessed through the reporting of adverse events, physical examination, recording of vital signs.

### Statistical analysis

The primary endpoint “time to response” is defined as the first time point in minutes after Dose 1 at which CGI-I score of 1 or 2 is achieved during the 120 min after the first dose. The comparison of time to response between the two treatment groups will be performed using a Wilcoxon rank-sum test with a two-sided significance level of 0.05. Patients without response within 2 h will be considered as non-responders and the ‘time to response’ as 4 h (2 h more than the maximum follow-up for this variable). The median difference (95% CI) will be estimated by means of the Hodges-Lehmann approach based on the Wilcoxon rank-sum (Mann-Whitney) distribution. Kaplan-Meier curves of time to response will also be presented by treatment group. Additionally, the Mantel-Haenszel log-rank test will also be conducted for sensitivity purposes.

Secondary analyses will be exploratory without formal sample size estimation. Safety and tolerability data will be presented descriptively.

A total sample size of 180 in each group will have approximately 90% power to demonstrate superiority of inhaled loxapine versus IM aripiprazole for the primary efficacy endpoint at a one-sided significance level of 0.025. If the interim analysis suggests that more than 30% additional patients are required at 0.025 one-tailed alpha level and approximately 90% power, it will be considered if it is feasible to include >30% additional patients. Since there will be neither stopping for overwhelming efficacy, nor is there a situation where the sample size will decrease based on the interim analysis results, the alpha level will be preserved.

## Discussion

Acute agitation in patients with psychiatric disorders including schizophrenia and bipolar disorder is a serious complication with the potential to escalate rapidly to aggression and physical violence [3, 5, 6]. Early recognition of the onset of acute agitation and intervention to rapidly calm the agitated patient without over-sedation are the main goals of the pharmacological management of such patients [3]. Antipsychotic medications are widely used in this setting and IM delivery provides a more rapid onset of effect compared with oral formulations. However, IM delivery of medication in this setting may not be optimal for patients already in an agitated state and may interfere with the therapeutic relationship between the patient and their carers [2]. Inhaled formulations have the potential to provide a more rapid onset of effect than IM injection. The medication is delivered through the lung with rapid transition to the systemic circulation via a non-coercive, non-invasive route of administration [3, 25]. Clinical evaluation of the inhaled antipsychotic loxapine has demonstrated an onset of therapeutic effect as early as 10 min after dosing, an effect that is maintained through 24 h among patients with acute agitation and schizophrenia or bipolar disorder [15, 16, 21, 26].

The PLACID trial will assess the efficacy and safety of inhaled loxapine compared with the IM antipsychotic, aripiprazole, in acutely agitated patients with schizophrenia or bipolar disorder. The PLACID trial is planned as a pragmatic trial close to the real world setting.

The CGI scale, which was developed as a brief, stand-alone assessment of the patient’s global functioning prior to and after initiating a study medication, will be used to assess the primary endpoint. This is a simple instrument widely used in a clinical practice and can be completed in less than a minute by an experienced rater [27] Even if not validated for agitation assessment, it was shown that there is a very good correlation between the CGI and PANSS-EC [28]. All raters will be trained in the proper CGI use to assess the agitation grade.

Both inhaled loxapine and IM aripiprazole are currently available in Europe for the treatment of acute agitation in patients with schizophrenia or bipolar disorder.

REMS (Risk Evaluation and Mitigation Strategy) is necessary with inhaled loxapine to mitigate the risk of bronchospasm. For this reason, only the centers with immediate access on site to supplies and personnel trained to manage acute bronchospasm will participate in this study. REMS is the strategy to manage a known or potential serious risk associated with a drug or biological product required by the US FDA (Food and Drug Administration).

The pharmacokinetic profiles (PK) of both drugs differ significantly and this is explained by the differences in the administration route. The peak plasma concentration of IM aripiprazole is achieved at 1 h, while for inhaled loxapine it is 2–3 min. Such a short time is explained by the fact that inhaled loxapine is directly absorbed into the blood stream with a PK profile similar to IV formulations. This study will prove if these differences in PK parameters correspond to clinical parameters, namely shorter time to response of inhaled loxapine.

Patients will be randomly assigned to treatment to avoid bias in terms of treatment allocation. The delivered treatment is necessarily open-label due to the different methods of administration. To avoid the issue of rater bias, drug administration and clinical evaluation will be carried out by different personnel, with the clinical rater blinded to treatment allocation and the randomization will be stratified by diagnosis to balance the groups. Patients will be instructed not to tell the treatment they receive.

## Conclusion

In conclusion, the prompt stabilisation of patients with schizophrenia or bipolar disorder presenting with acute agitation has the potential to facilitate a patient-centered management approach by maintaining the patient-physician relationship and minimising the need for physical or mechanical restraint or sedation [2, 3]. The PLACID study will allow, for the first time, the direct comparison of the timing of the onset of therapeutic effect of an IM antipsychotic, aripiprazole, and an inhaled antipsychotic, loxapine, for the treatment of acute agitation in patients with schizophrenia or bipolar disorder. The results of this trial have the potential to change future treatment guidelines and have a powerful impact on clinical practice.

## Additional file

Additional file 1: Ethics committees that approved the study: The contains the names and other relevant information of all the ethics committee that have approved the study. (XLS 59 kb)

## Abbreviations

CGI-I: Clinical global impression of improvement
CGI-S: Clinical global impression of severity
DSM: Diagnostic and statistical manual of mental disorders
IM: Intramuscular
TSQM: Treatment satisfaction questionnaire for medication
WHO: World Health Organization
